# Implantable Cardioverter-defibrillator Therapy in the Patient with a Left Ventricular Assist Device: Still a Black Box

**DOI:** 10.19102/icrm.2017.081205

**Published:** 2017-12-15

**Authors:** Travis Richardson, Christopher R. Ellis

**Affiliations:** ^1^Vanderbilt Heart and Vascular Institute, Nashville, TN, USA

**Keywords:** Heart failure, implantable cardioverter-defibrillator, left ventricular assist device, review

## Introduction

The use of implantable cardioverter-defibrillators (ICDs) has become a cornerstone in the therapy of patients with New York Heart Association (NYHA) classes II and III congestive heart failure, as randomized controlled trials have soundly established the lifesaving value of these devices.^1–5^ However, in patients with severe class IV NYHA symptoms or class D disease, the relative risk of sudden cardiac death compared with death from progressive pump failure decreases dramatically, and ICD therapy is not recommended in these individuals.^6,7^

More recently, though, the use of implantable mechanical left ventricular assist devices (LVADs) has changed the face of care for this group of patients. Since the risk of pump failure in this group is mitigated by LVAD therapy, ICD therapy has been recommended by the 2013 International Society for Heart and Lung Transplantation (ISHLT) guideline statement.^8^ Despite this recommendation, the exact role of ICD therapy, and the best way to program an ICD in this population, remains uncertain.

## ICD therapy in LVAD patients

The principal question remaining unanswered is whether or not ICD therapy provides a survival benefit in patients with an LVAD. To date, there has been no randomized prospective evaluation of ICD therapy in this population. Several retrospective evaluations have suggested a survival benefit for ICD therapy in patients with LVAD support,^9,10^ while others have not come to this conclusion.^11–14^ Two meta-analyses have been performed in recent years that may provide insight into the disparity of these results. One analysis performed by Vakil et al. examined six observational studies encompassing data from 937 patients, 93% of whom had LVAD therapy as a bridge to transplantation, 39% of whom had a continuous-flow (CF) device, and 38% of whom had an ICD in place. This analysis found a relative risk of 0.61 for all-cause mortality with a confidence interval of 0.46 to 0.82 (p < 0.01).^15^ The major concern with these data is their generalizability to modern LVAD therapy, where the vast majority of patients are implanted with CF devices and have pre-existing ICDs in situ. As such, there is concern that the survival trend observed in this meta-analysis is likely to be confounded by a higher overall intensity of care, or by a selection bias toward patients with a better prognosis in individuals with an ICD implanted.

A second analysis by Agrawal et al. only examined patients with continuous-flow LVAD devices, including data from 292 patients, 69% of whom had an ICD implanted.^16^ This analysis did not suggest a survival benefit associated with ICD use, and reported an odds ratio of survival of 1.47 with ICD therapy and a confidence interval of 0.42 to 1.47 (p = 0.45). Finally, this year, Younes et al. performed a propensity score-matched analysis involving patients with LVAD listed for heart transplantation in the United Network for Organ Sharing database between 2008 and 2015. In their investigation, 722 patients with an ICD were compared with 722 patients without ICD, and no association between decreased waitlist mortality and ICD use was found.^17^ In summary, while the largest analysis of available data suggests a survival benefit associated with ICD therapy in LVAD patients, more recent data that likely better represent “today’s” LVAD patient do not corroborate this conclusion. Thus, we are left with uncertainty regarding the benefit of ICD therapy in these patients and, certainly in some individuals (such as those with inappropriate shocks, ventricular tachycardia storm, or cardiovascular implantable electronic device-associated infection), indications that the ICD may do more harm than good.

## LVAD implantation in ICD patients

Though the role of ICD therapy remains in question, it is important to note that the majority of patients who undergo LVAD implantation already have an ICD in place preoperatively. This means that the question of proper programming in this population is possibly more germane to their care than the question of whether to implant an ICD or not. The clinical implications of ventricular arrhythmias (VAs) are seemingly clearer. In many cases, patients with LVADs who experience VA, or even prolonged asystole, will remain asymptomatic, or nearly so.^18–20^ A small, well-performed clinical study by the group at the Cleveland Clinic examined hemodynamic changes during defibrillation threshold testing among patients with LVADs in place who were undergoing ICD implantation. This study demonstrated a 32% reduction in LVAD flow during ventricular fibrillation, with return to normal flow with restoration of sinus rhythm.^21^ These findings imply that these arrhythmias do affect cardiac output, but also that LVAD therapy appears to make a life-threatening VA like ventricular fibrillation into one of non-emergent importance. With these data in mind, it seems most appropriate to think of VAs in the LVAD patient similarly to the way one might think of atrial arrhythmias in the unsupported patient with chronic heart failure. In both instances, these arrhythmias may lead to symptomatic low-output states, but in the vast majority of cases, the arrhythmia is not life-threatening.

**Figure 1:**
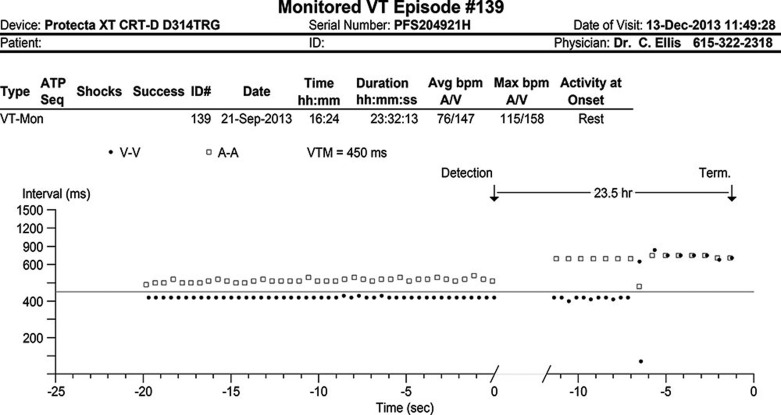
Intracardiac electrogram showing ventricular tachycardia sustained for 23.5 hours with spontaneous termination followed by an atrial pacing-biventricular pacing paced rhythm.

**Figure 2:**
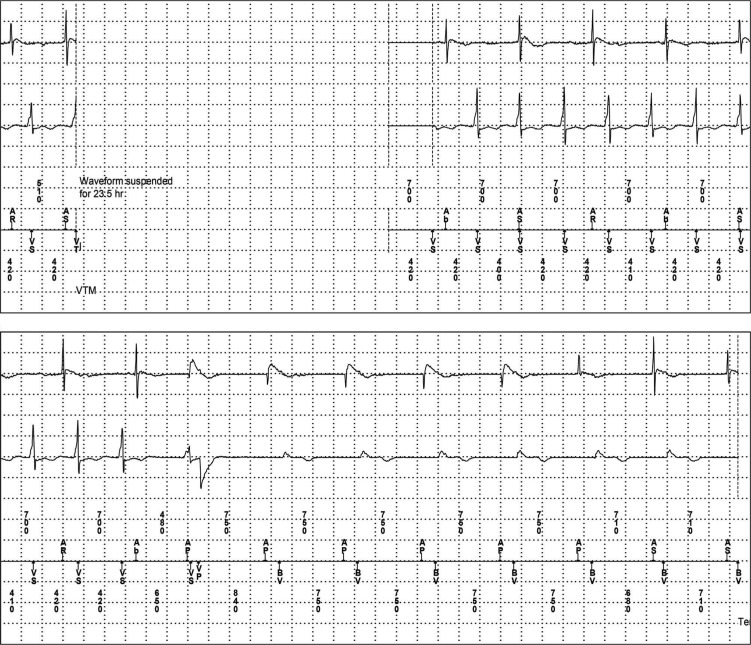
Intracardiac electrogram showing ventricular tachycardia sustained for 23.5 hours with termination a single premature ventricular contraction with VSR pacing.

Unlike in the patient without an LVAD, the tolerance of VAs for minutes to hours or even days, rather than seconds, is commonplace in those with LVADs **(Figures 1 and 2)**. As such, traditional ICD programming strategies in this group seem unlikely to be appropriate; indeed, one study found that implantation of a CF LVAD was associated with changes in the performance of pre-existing ICD, including the occurrence of ICD-related adverse events.^22^ Another suggested electromagnetic interference between LVADs and ICDs may ultimately necessitate ICD replacement.^23^ Unfortunately, device company firmware restrictions in ICDs do not truly allow for appropriate LVAD programming to be employed. Currently, the longest possible programmable detection interval prior to tachytherapy delivery is in the range of 30 seconds to one minute.

In light of this, one could argue that perhaps we need to change our approach to VAs in the LVAD population drastically. It seems reasonable, in the coming years, to investigate the role of a remote monitoring strategy for VA. Detection could prompt alerts to physicians or patients and perhaps urgent, rather than device-initiated, cardioversions could be arranged. This has the potential to reduce both hospitalizations and painful ICD shocks in the LVAD population. One common theme among previously published studies is that the best predictor of appropriate ICD therapy after an LVAD is implanted is the presence of sustained VAs prior to implantation (ie, secondary prevention population).^24^

Other questions that must be considered include the potential for and the management of infection in the setting of concomitant LVAD and cardiovascular implantable electronic device implantation,^25^ and the safety and efficacy of LVAD implantation done in conjunction with that of newer technologies, such as subcutaneous ICDs.^26^ The efficacy and benefits of cardiac resynchronization therapy device use in LVAD recipients also remains a topic of interest.^27,28^

## Conclusions

In summary, there are two important questions regarding ICD therapy in the LVAD population that remain unanswered: does ICD therapy improve survival in the LVAD patient, and how should existing ICDs be programmed in patients with LVAD therapy? Prospective, randomized investigations will be required to advance our understanding of ICD therapy in this growing population.
